# Genomic Analysis of Antimicrobial Resistance in *Pseudomonas aeruginosa* from a “One Health” Perspective

**DOI:** 10.3390/microorganisms12091770

**Published:** 2024-08-27

**Authors:** Celia García-Rivera, Carmen Molina-Pardines, José M. Haro-Moreno, Mónica Parra Grande, Juan Carlos Rodríguez, Mario López-Pérez

**Affiliations:** 1 Microbiology Department, Alicante Institute of Sanitary and Biomedical Research (ISABIAL), Alicante University General Hospital, 03010 Alicante, Spain; garcia_celriv@gva.es (C.G.-R.); parra_mongra@gva.es (M.P.G.); 2 Evolutionary Genomics Group, División de Microbiología, Universidad Miguel Hernández, 03550 Alicante, Spain; c.molina@umh.es (C.M.-P.); moreno.jose@umh.es (J.M.H.-M.)

**Keywords:** *Pseudomonas aeruginosa*, One Health, antimicrobial resistance, antibiotic resistance, pangenome, mobile integron

## Abstract

The “One Health” approach provides a comprehensive framework for understanding antimicrobial resistance. This perspective is of particular importance in the study of *Pseudomonas aeruginosa*, as it is not only a pathogen that affects humans but also persists in environmental reservoirs. To assess evolutionary selection for niche-specific traits, a genomic comparison of 749 *P. aeruginosa* strains from three environments (clinical, aquatic, and soil) was performed. The results showed that the environment does indeed exert selective pressure on specific traits. The high percentage of persistent genome, the lack of correlation between phylogeny and origin of the isolate, and the high intrinsic resistance indicate that the species has a high potential for pathogenicity and resistance, regardless of the reservoir. The flexible genome showed an enrichment of metal resistance genes, which could act as a co-selection of antibiotic resistance genes. In the plasmids, resistance genes were found in multigenic clusters, with the presence of a mobile integron being prominent. This integron was identified in several pathogenic strains belonging to distantly related taxa with a worldwide distribution, showing the risk of rapid evolution of resistance. These results provide a more complete understanding of the evolution of *P. aeruginosa*, which could help develop new prevention strategies.

## 1. Introduction

Bacterial antimicrobial resistance (AMR) is widely recognised as a major global public health threat in the 21st century [1]. While antibiotic discovery has saved millions of lives in recent decades, their overuse and inappropriate administration in human and veterinary medicine have led to a decline in their efficacy and the adaptation of bacteria to virtually all available treatments [2]. This has led to an increase in deaths caused by multidrug-resistant bacteria, longer hospital stays, and higher healthcare costs [3]. In 2022, the European Commission and the European Union (EU) Member States identified AMR as one of the top three priority health threats. It has been estimated that AMR directly causes 1.27 million deaths worldwide annually [4]. When deaths associated with multidrug-resistant microbial infections are taken into account, the number rises to approximately 5 million people. This makes AMR the third leading cause of death after cardiovascular disease and cancer [4]. Additionally, the World Health Organisation (WHO) has predicted that antibiotic resistance will cause around 10 million deaths per year by 2050, with costs to the global economy reaching up to USD 100 trillion [5]. The rapid emergence of mutations in bacteria when exposed to antibiotics has made these drugs unattractive to pharmaceutical companies, resulting in a halt to their development. The last generation of antibiotics was developed in the early 1960s [6], which is a cause for concern. Although multiple alternative approaches are being investigated, a reliable substitute for antibiotics has not yet been found [7].

AMR is not confined to humans or clinical settings. The advent of culture-independent methodologies, such as metagenomics, has led to the realisation that AMR is a multifaceted issue, entailing interactions between diverse bacterial populations that exert an influence on the well-being of humans, animals, and the environment [8]. While some classes of antimicrobials are reserved exclusively for human use, the majority are also employed in livestock, aquaculture, and agriculture. This results in the persistence of drug residues in the environment, which leads to the continuous emergence of new resistance mechanisms. It is crucial to acknowledge that the utilisation of antimicrobials in non-human contexts can exert a considerable influence on human health [9]. It is therefore essential to consider the risk of AMR and its transmission holistically at the animal–human–environment interface to delay the emergence of new resistance and preserve the efficacy of antibiotics until a new alternative is found. This approach is known as “One Health” [10]. The “One Health” approach, supported by coordinated surveillance and education, is essential for preventing antimicrobial resistance (AMR) and promoting global health. Organizations such as the FAO, the OIE, and the WHO have adopted this concept in their AMR action plans. These initiatives aim to prevent AMR through awareness programs, education on responsible antibiotic use, political commitment, and effective antimicrobial management [11].

In 2017, the WHO published a list of pathogens that are responsible for most hospital-acquired infections due to their resistance to antimicrobial agents. The ESKAPE pathogens are regarded as a category of particular importance within this comprehensive list [12]. ESKAPE is an acronym that includes the scientific names of six highly virulent and antibiotic-resistant bacterial pathogens: *Enterococcus faecium*, *Staphylococcus aureus*, *Klebsiella pneumoniae*, *Acinetobacter baumannii*, *Pseudomonas aeruginosa*, and *Enterobacter* spp. The acronym has recently been extended to ESKAPEE to also include *Escherichia coli* [13]. Understanding the resistance mechanisms of these bacteria is crucial for developing novel antimicrobial agents or other alternative tools to combat these public health challenges [14]. Among the priority antibiotic-resistant pathogens, *P. aeruginosa* is classified as a level 1 (critical) priority due to the increasing difficulty in treating infections caused by this bacterium, which can potentially be fatal [15]. This Gram-negative pathogen is opportunistic and responsible for severe infections with high morbidity and mortality rates in immunocompromised patients, particularly in hospital settings and Intensive Care Units (ICUs) [16]. It is the most common cause of chronic respiratory infections in patients with cystic fibrosis [17]. Due to the presence of efflux pumps and low membrane permeability, *P*. *aeruginosa* exhibits intrinsic reduced susceptibility to several antibacterial agents [18,19]. *P. aeruginosa* is a versatile bacterium that has been identified in a range of diverse ecological settings, including soil, water, plants, and human environments. In soil and water, it plays a significant role in the decomposition of organic matter and the breakdown of complex organic compounds, including pollutants. This contributes to nutrient cycling and bioremediation [16]. In plant environments, the bacterium can act as both a plant growth-promoting bacterium, producing substances that inhibit plant pathogens, and as a pathogen itself under certain conditions [20]. Moreover, *P. aeruginosa* plays a role in nitrogen fixation in certain ecosystems [21]. However, there is a lack of knowledge regarding environmental isolates of this microbe and their potential role as reservoirs of antibiotic resistance. Therefore, the objective of this study is to examine the evolutionary selection of niche-specific traits in clinical strains of *P. aeruginosa* in comparison with their environmental counterparts obtained from soil and aquatic environments. This will be achieved through a pangenomic approach. It is of paramount importance to ascertain the relationship between the environmental reservoirs of *P. aeruginosa* resistance and its mode of transfer in order to develop effective strategies for the prevention of infections caused by multidrug-resistant bacteria.

## 2. Materials and Methods

*P. aeruginosa* strains. The 749 *P. aeruginosa* used in this study were downloaded from the Pseudomonas Genome Database [22]. Of the total number of isolates, 591 were derived from human sources, 103 from aquatic environments (including marine and freshwater), and 55 from soil. Genomes were selected based on metadata indicating their environmental source, and those with more than 100 contigs were excluded from the analysis. The genomic features of these strains are detailed in the Table S1. For each genome, coding DNA sequences were predicted with Prodigal v2.6 using -*a output.proteins* -*d output.genes* -*c* -*p meta parameters* [23]. Then, we used DIAMOND v2.0.6 [24] (blastp *-sensitive -max-target-seqs 1 -evalue 1-e5 –block-size 12.0 -index-chunks 1*) to annotate the sequences against the non-redundant protein sequences from the NCBI database (NCBI’s NR). The inferred function was predicted comparing protein sequences against COG [25] and TIGFRAM [26] databases using HMMscan v3.1b2 [27]. For statistical analysis, quantitative variables were expressed as the mean ± standard deviation (SD) and compared by the unpaired t-test. Statistical analysis was performed using SPSS version 15.0 (SPSS, Inc., Chicago, IL, USA). All *p*-values were two-sided, and *p* < 0.05 was considered statistically significant.

Phylogenetic characterization. To establish the divergence of the genomes, average nucleotide identity values (ANI) among genomes were calculated using the FastANI program [28]. The dRep software was used to dereplicate highly identical genomes (ANI > 99%) [29]. Phylophlan3 was used to establish the phylogenomic classification using the following parameters: -d phylophlan -t a –diversity high –accurate -f supermatrix_aa.cfg [30]. We modified the program to use IQ-TREE [31] with the LG+F+G4 amino acid model and an ultra-fast bootstrap of 1000 replicates [32]. The resulting phylogenomic tree was edited using iTOL [33].

Pangenome analysis. To analyse the prevalence of gene families within the *P. aeruginosa* species, we employed PPanGGOLiN to classify gene families into persistent, shell, and cloud partitions [34]. The genes constituting each partition were functionally annotated against the SEED subsystems database [35], using DIAMOND v2.0.6 (blastp option, top hit, ≥ 50% identity, ≥ 50% alignment length, E-value < 10^−5^). Only matches with E < 0.001 and alignment length > 0.5 for both subject and query were taken into account. The MEGARes database [36] and the virulence factor database [37] were used to locate antimicrobial resistance genes and virulence factors, respectively.

The *P. aeruginosa* mobilome. Identification of plasmids and prophages within the genomes was carried out using Plasmer [38] and VIBRANT v1.2.1 [39] software, respectively. After manual curation, the predicted protein-coding genes in both mobile elements were taxonomically and functionally annotated against the non-redundant protein sequences from the NCBI database (NCBI’s NR) using DIAMOND v2.0.6 [25]. Putative endolysins were extracted following the method described by Fernández-Ruiz et al. [40]. The predicted proteins from each prophage were compared against a curated database of endolysins using DIAMOND v2.0.6. Matches were classified as putative endolysins if the match had >50% identity, covered at least 30% of the query sequence, the alignment was at least 50 aa long, and the E value was at least 10^−3^.

## 3. Results and Discussion

To investigate the genomic differences between *P. aeruginosa* strains with different lifestyles (i.e., free-living to pathogenic), a total of 749 *P. aeruginosa* genomes were downloaded from the Pseudomonas Genome Database [22]. This database integrates high-precision genome-scale genomic sequences as well as manually curated metadata. Given the significant bias in the databases towards potentially pathogenic strains, we obtained 591 strains isolated from humans and 158 from environmental sources, including 103 from aquatic and 55 from soil. The genomic features of the genomes used in this study are available in Table S1. Despite belonging to the same species, analysis of their genomic characteristics revealed statistically significant differences among the three groups. The aquatic representatives exhibited a larger mean genome size of 6.6 Mb (SD, ±0.27) in comparison to the other two environments, with clinical and soil having means of 6.5 Mb (SD, ±0.22) and 6.4 Mb (SD, ±0.17), respectively (*p*-value < 0.05) (Table S1).

**Figure 1:**
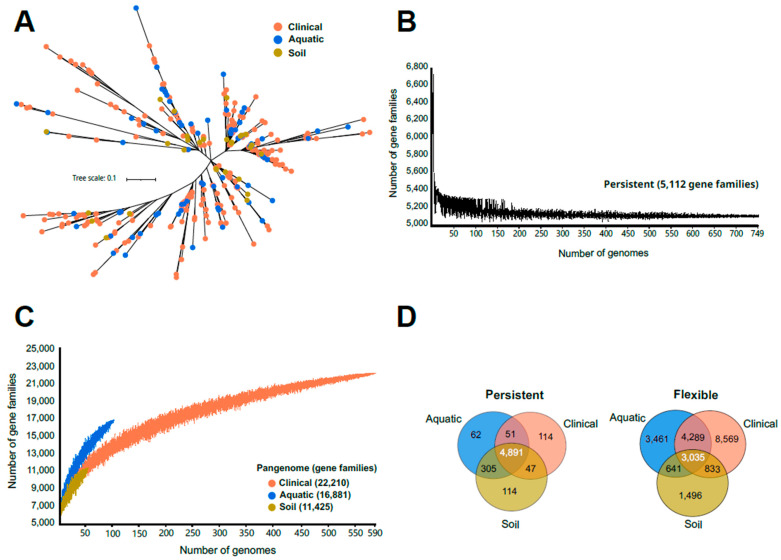
(**A**) Phylogenomic tree of dereplicated *P. aeruginosa* genomes (ANI > 99%). (**B**) Core genome size accumulation of the 749 *P. aeruginosa* strains of the three environments (clinical, aquatic, and soil). (**C**) Pangenome size accumulation of the 749 *P. aeruginosa* strains of the three environments (clinical, aquatic, and soil). (**D**) Venn diagram representing the relationship between the gene families of the persistent and flexible genome across the three environments.

Phylogenomic characterization. To obtain a characterization of the genomic diversity within the recovered *P. aeruginosa*, a whole-genome phylogenomic tree was performed using only dereplicated genomes (ANI > 99%) to avoid redundancies (#332 genomes; Figure 1A). The supplementary material includes a phylogenomic analysis of all genomes (Figure S1). The analysis demonstrated that the origin of *P. aeruginosa* strains was not correlated with the topology of the tree (Figure 1A). Phylogenomic analysis and ANI-based genome clustering enabled the genomes to be classified into 10 clusters with 99% identity, and only three clusters for the 98% ANI threshold. These results indicate that *P. aeruginosa* exhibits low intra-population sequence diversity, as the accepted cut-off for species membership is ANI 95%. Previous studies have indicated that the colonisation of new habitats in this species does not appear to necessitate the acquisition of new mutations, which would otherwise lead to population homogenisation [41]. The genomic homogeneity within the population and the environmental distribution unbiased by phylogenomic analysis suggest two possible evolutionary trends in the genome: a broad metabolic versatility of the core genome or a high genetic diversity of the flexible genome. This could influence the epidemiology of this microbe, as the same genomic background could mean that all strains, regardless of the origin from which they were isolated, would be potentially pathogenic.

***Pseudomonas aeruginosa* pangenome.** Despite the absence of a phylogenetic relationship between strains and their respective environments, we decided to compare the genomic diversity of strains from each environment individually, using pangenomic analysis. Each of the pangenomes was divided into a persistent genome (also referred to as core genome), which comprises gene families present in almost all the genomes (at least 95%), and a flexible genome, which was divided into shell and cloud genome; shell genome consisted of gene families present in at least 15% but less than 95% of the strains and cloud genome referred to the set of gene families that were present in less than 15% of the genomes. Figure 1B shows that the persistent genome of all genomes in the study rapidly reached a plateau, while the pangenome curve of the strains from the three environments did not saturate, indicating an open pangenome in all cases. Considering all the genomes recovered in the study (regardless of the environment), the persistent genome comprised 5112 gene families, representing on average 91.7% of a *P. aeruginosa* genome. When the entire pangenome (26,590 gene families) was considered, the persistent genome represented 19%. The pangenome data for each environment revealed a total of 22,210, 16,881, and 11,425 gene families for the clinical, aquatic, and soil environments, respectively (Figure 1C).

In order to assess the potential adaptations to each environment, it was necessary to analyse the pangenome of each dataset separately. While the persistent genome of the 749 strains comprised 5112 gene families, analysis of the pangenome considering each environment independently revealed that the number of gene families shared between them was reduced to 4891 (on average, 87.7% of a *P. aeruginosa* genome) (Figure 1D). The Venn diagram of the different persistent genome revealed that the non-clinical environments (aquatic and soil) shared the greatest number of gene families, with a total of 305. However, each environment exhibited some unique gene families, with 114 in human and soil and 62 in aquatic. This indicates that, despite the absence of a correlation between phylogeny and the origin of the strains, specific gene families within each dataset may be implicated in the adaptation of the strains to their respective environments, despite their classification as belonging to the same species. However, when considering the environments separately, the persistent genome represents a high percentage of the genome (87.7%) compared to other ESKAPE pathogens such as *Enterococcus faecium*, *Staphylococcus aureus*, *Klebsiella pneumoniae*, or *Acinetobacter baumannii* [42,43,44,45].

A comparison of the flexible genome (shell and cloud) across environments revealed a total of 3035 gene families in common. In this case, the environments sharing the highest number of gene families were clinical and aquatic, with a total of 4289. Additionally, each environment exhibited a subset of gene families that were specific to that environment, in proportion to the diversity and abundance of strains within each dataset. The total number of unique gene families in clinical, aquatic, and soil environments was 8569, 3461, and 1496, respectively (Figure 1D).

**Functional analysis of the pangenome.** A functional analysis was conducted on gene families belonging to the persistent genome that were unique to each environment. This analysis was performed using the SEED subsystems database [35]. The data showed that the gene families unique to the persistent soil genome were the only ones with activity related to sulphur metabolism (arylsulphatase, al-kanesulphonate transport system permease protein, ABC-type nitrate/sulphonate/bicarbonate transport systems, periplasmic components). However, they lacked functions related to DNA and fatty acid metabolism, lipid and isoprenoid metabolism, motility and chemotaxis, nitrogen metabolism and protein metabolism that were present in the clinical and aquatic strains (Table S2). In the case of strains belonging to aquatic environments, the persistent genome exhibited a notable prevalence of membrane transporters, a category of genes that is less abundant in soil and human environments. This phenomenon is likely attributable to the oligotrophic nature that characterises aquatic environments, which has facilitated the enrichment of the persistent genome with a greater number of nutrient uptake systems.

**Figure 2:**
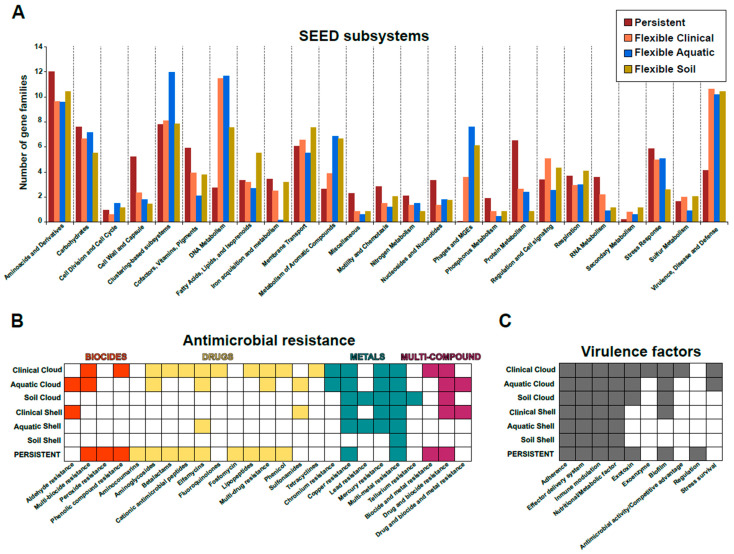
Pangenome analysis and carbon utilization of *Pseudomonas aeruginosa* strains. (**A**) Functional characterization of the pangenome of the three environments using the SEED subsystems database. (**B**) Distribution and classification of antimicrobial resistance categories in the different pangenome partitions. (**C**) Abundance and distribution of virulence factors in the pangenome partitions of the three environments.

The proportion of gene families in the persistent genome that could be assigned to a SEED category was 58%, while for the flexible genome it was only 23%, 19%, and 23% for the clinical, aquatic, and soil environments, respectively. These data demonstrate the substantial amount of information that remains undiscovered due to a lack of annotation. As expected, the persistent genome shared by all environments was found to be enriched in categories related to metabolic processes, such as amino acid biosynthesis, cell wall, RNA metabolism, protein metabolism, and nucleoside/nucleotide metabolism. In contrast, the flexible genome was found to share several categories such as presence of prophages and transposable elements, secondary metabolism and virulence, disease, and defence (Figure 2A). The function related to clustering-based subsystems was present in higher proportion in the flexible genome of the aquatic strains. A greater proportion of functions related to DNA metabolism was observed in the flexible genome of strains of human and aquatic origin compared to the soil environment. It is also notable that a greater proportion of gene families associated with the category of phages, prophages, and transposable elements was identified in strains of aquatic origin. The flexible genome of soil strains exhibited a higher proportion of functions related to the metabolism of fatty acids, isoprenoids, and phospholipids within the category of fatty acids, lipids, and isoprenoids (Figure 2A). In contrast, clinical strains exhibited enhanced triacylglyceride metabolism. Furthermore, a greater proportion of gene families within the cell regulation and signalling category was observed in these human strains. This may be attributed to the necessity for heightened regulation in a more active environment, with a markedly diverse microbiome associated with other environments, coupled with host defences and the presence of continuous nutrients.

**Antimicrobial resistance genes.** The presence of antimicrobial resistance genes (ARGs) in the different pangenome partitions was analysed using the MEGARes 2.0 database [36]. A threshold of 70% (BLASTP) was employed to identify a total of 67 ARGs in the persistent genome, representing approximately 1.3% of the total genes. Four distinct classes of ARGs were identified, including multi-compound (41.8%), drug resistance (34.3%), biocides (14.9%), and metal (9%) (Figure 2B). The category of “drug resistance” encompasses several different types of resistance, including beta-lactams resistance, aminocoumarins, elfamycins, lipopeptides, cationic antimicrobial peptides, fosfomycin, and aminoglycosides. Therefore, regardless of the environment, the species has a high degree of intrinsic resistance to several antibiotics. These results may have an evolutionary implication that highlights the serious problem of resistance. Notably, our findings reveal that this species exhibits a significantly higher degree of intrinsic resistance compared to other ESKAPE pathogens, which generally display a greater proportion of their resistances in the flexible genome rather than the core genome [46]. For example, in the genus of other ESKAPE bacteria, which are known to be environmental bacteria as well as important nosocomial (hospital-acquired) pathogens such as Acinetobacter, the relevant role of plasmids in the spread of AMR has been demonstrated [47,48]. The fact that antibiotics have been in the environment for decades may be causing some of the genes responsible for resistance to become part of the species’ persistent genome. If this is the case, this adaptation represents an important evolutionary step in the fight against resistance and warrants further investigation.

In terms of the two flexible genome partitions, the number of ARGs was low in the “shell” genome, with 19 ARGs identified in soil and clinical strains, while only one ARG was detected in the aquatic environment. In this pangenome partition, the predominant category was metal resistance across all environments. The cloud genome exhibited greater diversity, with a total of 83 gene families (0.6%), 26 (0.26%), and 23 (0.42%) associated with resistance in clinical, aquatic, and soil, respectively, being identified. The number of ARGs in the “cloud” genome was greater than in the “shell” genome. In this flexible genome partition, strains derived from the environment exhibited a higher proportion of metal resistance, while strains of clinical origin displayed a higher number of genes within the drug category. For instance, resistance to fluoroquinolones, beta-lactams, tetracyclines, and lipopeptides was observed exclusively in the “cloud” genome of clinical isolates. The presence of resistance to sulphonamides was observed exclusively in strains of human and aquatic origin. Consequently, in clinical strains, the flexible genome confers resistance to tetracyclines and fluoroquinolones. However, in soil and water strains, the flexible genome does not result in the emergence of new drug resistances beyond those already presents in the persistent genome. Detailed information of all antibiotic resistances found in both pangenome compartments in this study are available in Table S3.

In the “shell” genome, metal resistance genes were the most prevalent in all environments. In the environment, bacteria are not only subjected to androgenic stresses resulting from the presence of pharmaceuticals, but also to contamination by heavy metals. Therefore, bacteria have evolved a diverse array of resistance mechanisms against these compounds, which are widely distributed in the flexible genome of environmental strains [49]. Indeed, numerous studies have employed bacteria for the bioremediation of heavy metal-contaminated environments [50]. This has significant implications for the “One Health” process, as numerous studies have demonstrated that this heavy metal contamination can influence the maintenance and proliferation of ARG resistance through co-selection mechanisms [51].

**Virulence factors.** Next, the presence of virulence factors was analysed utilising the virulence factor database [37] with a threshold of 70% identity (BLASTP). A total of 339 virulence-associated genes were identified in the persistent genome of all the strains (representing 6.6% of the total) (Figure 2C). The categories with the highest representation were adherence, effector delivery system, immune modulation, nutritional/metabolic factor, exotoxin, biofilm, and regulation. In terms of the flexible genome, the “shell” genome was found to contain a total of 128 (3.7%), 80 (5%), and 62 (9.8%) gene families from clinical, aquatic, and soil strains, respectively. Although the relative abundance of these categories differed among environments, all environments exhibited the same categories, apart from the clinical environment, which also included the antimicrobial activity/competitive advantage category. A comparable proportion of genes associated with virulence factors was identified within the “cloud” genome, with a prevalence of approximately 1% across all environments. In contrast to the shell genome, all environments exhibited a virulence factor related to biofilm formation and exotoxin production. Furthermore, the aquatic and clinical strains showed a shared stress survival category. The “cloud” genome of human isolates exhibited the antimicrobial activity/competitive advantage category previously observed in the “shell” partition, with the addition of the exoenzyme category.

***Pseudomonas aeruginosa* mobilome**. Horizontal gene transfer represents a pivotal mechanism for the dissemination of resistance genes. Indeed, in a previous study, we demonstrated the significance of integrative and conjugative elements (ICE) in the dissemination of a plethora of antibiotic-resistant genes in *P. aeruginosa* [52]. For this reason, the present study was designed to examine the potential contributions of two additional mobile genetic elements, namely plasmids and prophages, to the transfer and dissemination of ARGs between environments.

***Plasmids***. A total of 85 plasmids were obtained, comprising 58 clinical, 19 aquatic, and 8 soil environments. The GC content of these plasmids ranged from 54.82% to 64.12% (with an average of 58.76%). The largest plasmid had a length of 468,631 bp, while the smallest plasmid was 14,918 bp. Of the total 85 plasmids, nine were identified as repeated plasmids (with a minimum of identity and coverage of 90%). All repeat plasmids were found within genomes from the same environment, except for one, which was found to be duplicated in both the clinical (AU10272-C67) and aquatic (AUS221-C4) environment. The ANI of the genomes containing these identical plasmids was 98.7%, indicating that they are non-clonal strains. The plasmid was found to contain genes encoding resistance to multiple antimicrobial agents, which are associated with multi-drug ABC efflux pumps. It has been demonstrated that plasmids can be transmitted between different bacterial species. However, the mechanisms by which plasmids are transferred between different environments remain poorly understood. This finding provides evidence for the existence of identical plasmids in both different strains and diverse environments. This is of significant importance for understanding the natural exchange of genes between bacteria, including those conferring resistance to antibiotics, which continue to pose a global health threat.

The resistance genes present within these plasmids were subjected to further investigation. A total of 84 ARGs were identified in 22 plasmids (accounting for 26% of all plasmids), with an average of four resistance genes per plasmid. The plasmids containing the greatest number of resistance genes, with a total of nine genes, were GOM1-C20 and TNCF-23M-C8. These plasmids were isolated from strains collected from clinical and human environments. No ARGs were identified in the plasmids from the soil-derived strains. Most of the proteins identified in both human and aquatic environments were related to metal resistance, followed by drug resistance. In clinical strains, and likely due to the enrichment bias observed in genome databases derived from antibiotic resistance studies, the plasmids exhibited gene resistance to aminoglycosides, tetracyclines, fluoroquinolones, sulphonamides, phenicol, and beta-lactams. In contrast, in the aquatic environment, resistance genes against aminoglycosides were the only ones identified. Conversely, a total of ten proteins (identity 70%) related to virulence factors were identified. These proteins were identified exclusively on plasmids of clinical origin within two categories, effector delivery systems and nutritional/metabolic factors.

**Figure 3:**
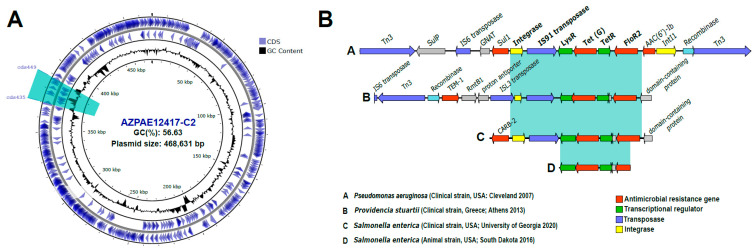
(**A**) Schematic representation of plasmid pAZPAE12417-C2 (436 Kb). The highlighted region corresponds to the location of the integron. (**B**) Genomic alignment of mobile genetic elements containing the same resistance gene cassette (>95% identity) as found within the AZPAE12417-C2 plasmid integron (**A**) in members of the orders Pseudomonadales and Enterobacterales.

Genomic analysis of the plasmids with multiple resistances revealed that, in one of them (pAZPAE12417-C2-436 Kb), multiple ARGs were concentrated in a region corresponding to an integron (Figure 3A). These non-chromosome-associated integrons are known as mobile integrons [53]. The ARGs identified within the integron were a tetracycline efflux MFS transporter, a sulphonamide-resistant dihydropteroate synthase, an AAC (6′)-Ib family aminoglycoside 6′-N-acetyltransferase, and the chloramphenicol/florfenicol efflux MFS transporter, FloR2. To analyse the dispersal capacity of this class of elements, a search for this integron was conducted in the NCBI non-redundant database. The results demonstrated the presence of the same integron and the associated ARGs, with a high identity (>95%), in microbes belonging to different orders within the class Gammaproteobacteria (Figure 3B). The *P. aeruginosa* strain containing this integron was obtained in 2007 from a cystic fibrosis patient in the USA [54]. However, the same mobile element was also recovered in other clinical and animal strains isolated worldwide. These include *Providencia stuartii* (clinical strain; Greece 2013), *Salmonella enterica* (clinical strain; USA, University of Georgia 2020), and *S. enterica* (animal strain; USA, South Dakota 2016). The co-occurrence of two mobile genetic elements, such as a plasmid and an integron, in disparate taxonomic species with high identity suggests the ease with which these elements can traverse taxonomic and geographical boundaries. Moreover, the integron enables not only the dissemination of ARGs but also their expression. This suggests that there is a significant possibility of dispersal and horizontal transfer of these elements between different environments, which could accelerate the evolution of resistance. In light of these considerations, there is a compelling need to develop effective multidisciplinary prevention and control strategies at both the clinical and environmental levels.

***Prophages***. A total of 2479 prophages were obtained, with GC contents ranging from 34.9% to 67.9% (with an average of 62.5%). The largest prophage was 105,638 bp, while the smallest was 1126 bp. A total of 221 lytic (9%) and 2258 lysogenic (91%) prophages were identified. The mean number of prophages per genome in each environment was three for the clinical isolates, but only one for both the aquatic and soil environments. The prophages were grouped into 709 clusters with an ANI value of 90%. A total of 454 prophages were identified only in a single genome. However, we found some examples where the same prophage was identified in strains from all three environments. The analysis of prophage-associated resistance genes showed the presence of 56 ARGs (0.06% of all prophage-encoded proteins). Most of the proteins identified in all environments were related to drug resistance. In strains derived from clinical isolates, resistance to fluoroquinolones and multidrug efflux pump function was identified. In strains from aquatic environments, we observed resistance to fluoroquinolones and to class D beta-lactams, which was not observed in strains from clinical samples. The strains from the soil exhibited resistance only to fluoroquinolones.

A total of 76 related proteins were identified within the prophages in terms of virulence factors. The human and aquatic environments exhibited several commonalities in their virulence factors, including those related to the effector release system, immune modulation, nutritional/metabolic factors, and regulation. Furthermore, in clinical isolates, we also identified virulence factors related to adhesion. In the soil environment, no virulence factors were identified in the prophages. The annotation of the prophages also revealed the presence of the zonular occludens toxin in 18 of them. The toxin was identified in 12 prophages derived from human isolates, five of which were isolated from aquatic environments and in one from soil. This toxin is a virulence factor that has been observed in other microorganisms, including *Vibrio cholera*, whose function is to increase the permeability of the intestinal epithelium [55], which would favour the bacteria in the infection process.

**Alternative to antimicrobials (endolysins).** One of the most significant challenges in microbiology is the management of infectious diseases caused by multidrug-resistant bacteria, due to the lack of effective treatments against them. Endolysins are enzymes produced by bacteriophages whose biological function is to hydrolyse bonds in the bacterial cell wall [56]. Consequently, they have become an increasingly popular complement to antibiotics for the treatment of infections caused by multidrug-resistant bacteria. A total of 1820 endolysins were identified within the prophage annotation. The endolysins were clustered at 90% identity, resulting in a total of 51 clusters. The functional classification revealed the existence of seven major functional clusters, including the glycoside hydrolase 19 (GH19) (51.4%), phage lysozyme (18.9%), chitin catabolic process (13.5%), bacteriophage lysis protein Rz (8.1%), host cell virus cytolysis (2.7%), lysozyme (2.7%), and N-acetyl muramidase (2.7%). The GH19 family is a bifunctional group, encompassing both chitinase and lysozyme activities. Except for phage lysozyme, which is more prevalent in the aquatic environment, all other activities were localised in prophages obtained from clinical settings. The high prevalence of these endolysins within the species suggests that they may play a significant role in the host and could be a promising candidate for the development of new antibacterial therapies against Gram-negative bacteria.

**Limitations of the study.** One of the principal constraints confronting “One Health” approaches such as this study, which endeavours to address health issues through a multidisciplinary and comprehensive perspective encompassing human, animal, and environmental health, is the preponderance of clinical strains in databases. A significant number of studies give precedence to clinical strains, given their greater accessibility through medical and hospital records. This may result in a biased understanding of antibiotic resistance, as it may not accurately reflect resistance profiles in the environment or in animals. The sampling and study of environmental strains is more challenging due to the diverse and less controlled nature of their habitats. This consequently creates a deficit in our understanding of the resistance genes present in non-clinical settings. Furthermore, studies that do not sufficiently incorporate environmental strains may fail to identify pivotal connections between environmental and clinical resistance mechanisms, impeding the formulation of comprehensive control strategies. For these reasons, and despite the existence of this bias in databases in general, it is essential to commence studying the contribution of environmental reservoirs of resistance to the overall burden of antibiotic resistance, as well as the pathways of transmission of resistance from the environment to humans and animals. In order to address these limitations in the future, it is necessary to adopt a more balanced approach that gives equal importance to environmental strains in antibiotic resistance research. This can be achieved through the implementation of improved sampling methods, interdisciplinary collaborations, and integrated surveillance systems that cover human, animal, and environmental health sectors in an equitable manner.

## 4. Conclusions

In conclusion, the “One Health” approach is of paramount importance for the study of resistance in *P. aeruginosa*, given that it is not only a human pathogen, but also affects animals and persists in diverse environmental reservoirs, such as soil and water. The high percentage of persistent genome within the species, the lack of correlation between phylogeny and origin of isolation, and the intrinsic resistance to a wide variety of antibiotics suggest that the species has high pathogenic and resistance potential regardless of the reservoir. This underlies the difficulty of its treatment and requires special attention for the development of new therapeutic strategies and a thorough understanding of its resistance pathways.

In the flexible genome, environmental strains exhibited an enrichment of metal resistance genes, which could act synergistically, allowing co-selection of antibiotic resistance genes. This, in turn, exacerbates the spread of AMR, particularly in mobile genetic elements. Only a quarter of the plasmids contained resistance genes, but the majority were present in multiple gene clusters. Prophages, which were much more abundant than plasmids, also contained genes directly conferring antibiotic resistance. We found examples of the same plasmid and prophage in different environments, which provides evidence of a connection and dispersion of these elements between environments. Most notable was the presence of a mobile integron within a plasmid that carried several associated resistances. Moreover, this integron was identified in multiple pathogenic strains belonging to distantly related taxa with a global distribution, thereby increasing the risk of rapid resistance evolution.

The findings of this study indicate that resistance management in *P. aeruginosa* may pose a significant challenge due to the organism’s metabolic versatility and the presence of multiple intrinsic resistances. However, this phenomenon is not exclusive to this species. The presence of this *P. aeruginosa* in multiple environmental reservoirs indicates the potential for the transfer of resistance genes to other human pathogens, which could further exacerbate the public health concern. Such gene flow has the potential to give rise to novel resistant strains within clinical settings, thereby complicating treatment options and increasing healthcare costs. Future research should prioritise the development of targeted strategies to mitigate the spread of resistance, as opposed to the emergence of new resistant strains as well as to explore the interactions between mobile genetic elements and their role in the spread of resistance genes in different environments. The practical applications of these findings should extend beyond the hospital setting to include increased surveillance of environmental reservoirs and stricter control measures in both clinical and agricultural settings, with the aim of preventing the spread of resistant strains. Any action against this threat must be multidisciplinary. This encompasses the implementation of enhanced antibiotic stewardship across all sectors, with the objective of reducing overall antibiotic utilisation and preventing the inadvertent release of antibiotics into the environment. Additionally, there is a need to reinforce environmental surveillance systems to monitor antibiotic resistance in *P. aeruginosa* and other pathogens. This integrated strategy is of paramount importance for the protection of public health and the long-term efficacy of antibiotics. Furthermore, efforts should be made to further the genomic analysis of environmental strains in addition to pathogenic strains.

## Data Availability

All sequences in this work as well as metadata have been obtained from the Pseudomonas Genome Database (https://www.pseudomonas.com/).

## Supplementary Materials

The following supporting information can be downloaded at: https://www.mdpi.com/article/10.3390/microorganisms12091770/s1, Figure S1: Maximum likelihood phylogenomic tree of the Pseudomonas aeruginosa genomes isolated in this study, Table S1: Genomic features of *Pseudomonas aeruginosa* strains used in this study; Table S2: Functional comparison of single gene families in each environment using the SEED database. Table S3: Antimicrobial resistance comparison of single gene families in each environment using the MEGARes database.
