# Supplementary figures and images for: Genomic Analysis of Antimicrobial Resistance in Pseudomonas aeruginosa from a “One Health” Perspective
Source: Microorganisms. 2024 Aug 27;12(9):1770. doi: 10.3390/microorganisms12091770 (PMC11433808; doi:10.3390/microorganisms12091770)

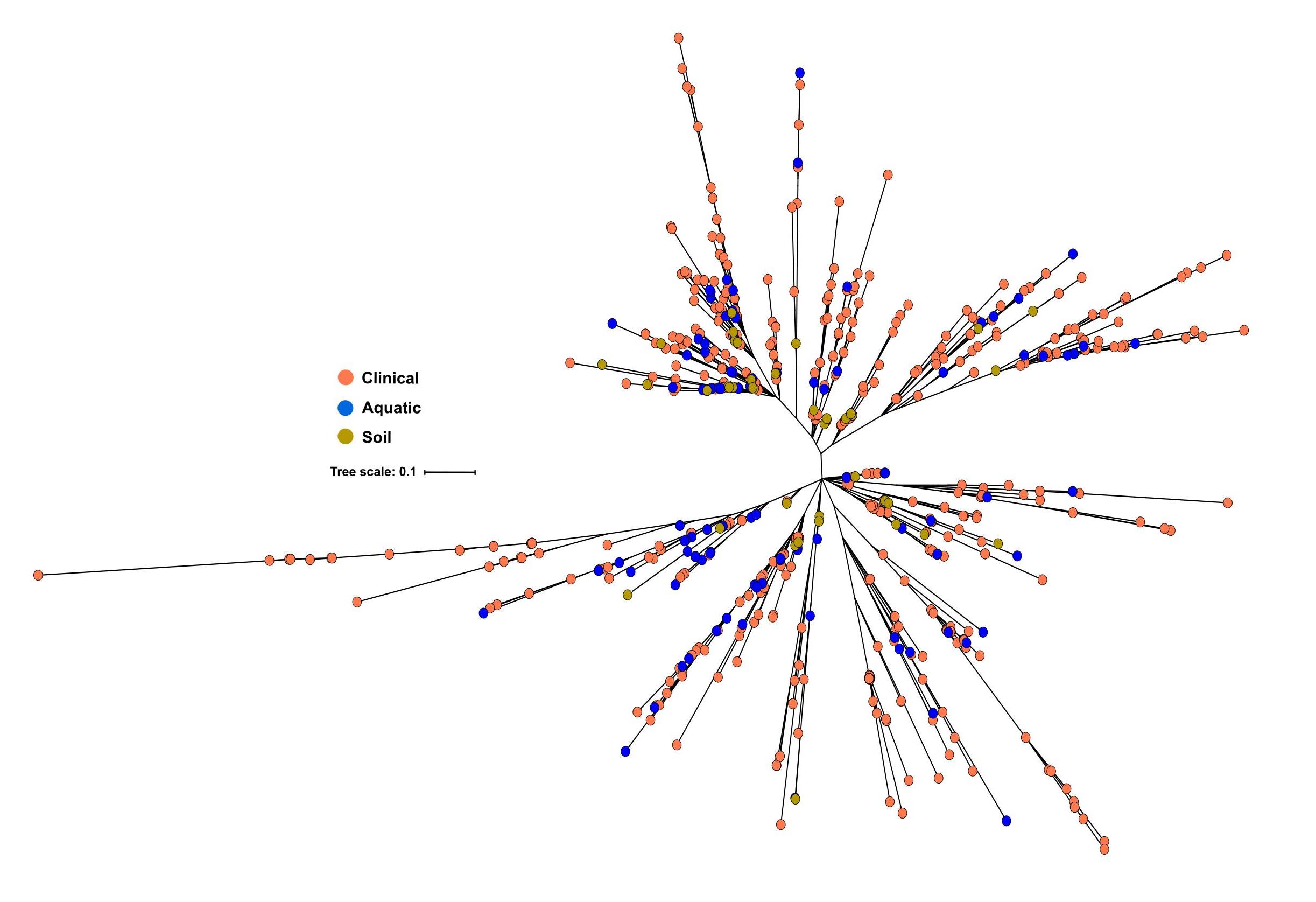

Supplement: Supplementary file 1 [file microorganisms-12-01770-s001.zip › Figure S1.jpg]
